# Foldable glycoprobes capable of fluorogenic crosslinking of biomacromolecules†
†Electronic supplementary information (ESI) available: Experimental section, additional figures and spectral copies. See DOI: 10.1039/c6sc02366e



**DOI:** 10.1039/c6sc02366e

**Published:** 2016-07-12

**Authors:** Kai-Bin Li, Na Li, Yi Zang, Guo-Rong Chen, Jia Li, Tony D. James, Xiao-Peng He, He Tian

**Affiliations:** a Key Laboratory for Advanced Materials & Institute of Fine Chemicals , School of Chemistry and Molecular Engineering , East China University of Science and Technology , 130 Meilong Rd. , Shanghai 200237 , PR China . Email: xphe@ecust.edu.cn; b National Center for Drug Screening , State Key Laboratory of Drug Research , Shanghai Institute of Materia Medica , Chinese Academy of Sciences , 189 Guo Shoujing Rd. , Shanghai 201203 , PR China . Email: jli@simm.ac.cn; c National Center for Protein Science Shanghai , Shanghai Institutes of Biological Sciences , Chinese Academy of Sciences , Shanghai 200031 , PR China; d Department of Chemistry , University of Bath , Bath , BA2 7AY , UK

## Abstract

Small-molecular probes capable of monitoring and interfering with the activity of biomacromolecules – such as polysaccharides, nucleotides and proteins – are of paramount importance to the advancement of life science. However, such probes that can detect and simultaneously modulate the construction of biomacromolecules are elusive. Here we report a fluorogenic, foldable glycoprobe that can recognize and assemble a protein receptor in a synchronous fashion. The glycoprobe synthesized by introducing a glycoligand (for protein recognition) to a bola-type bis-fluorophore conjugate shows a “self-shielded” fluorescence in the folded state. Association with a receptor protein rapidly unfolds the probe, releasing a fluorophore capable of crosslinking the proteins – as determined using small-angle X-ray scattering – thereby producing a unique fluorescent supramolecular construct. We have demonstrated the use of the foldable glycoprobe in order to track the endocytic cycle of a transmembrane receptor.

## Abstract 2

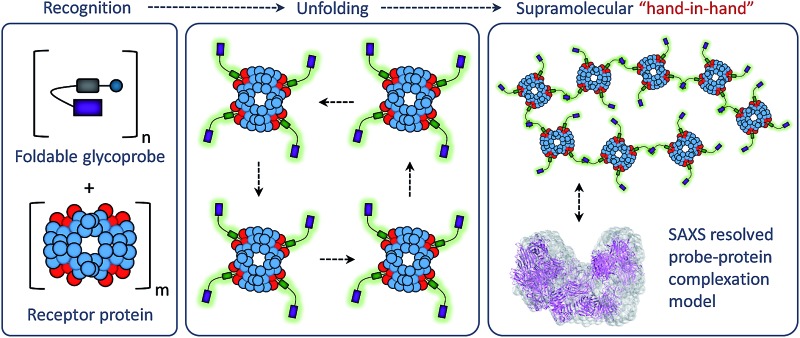
We demonstrate a foldable, fluorogenic glycoprobe that can recognize and simultaneously crosslink a receptor protein to form unique supramolecular bio-ensembles.

## Introduction

The ability to effectively identify and control the function of natural biomacromolecules, including nucleotides, proteins and polysaccharides, is of paramount importance to the advancement of life science. Indeed, the past few decades have seen the development of a variety of chemical probes to detect biomolecular targets and therapeutic agents to suppress their activities.^1–5^ However, molecular tools that can simultaneously monitor and modulate the activity of biomacromolecules have been elusive.

Receptor proteins are a class of macromolecules closely related to a number of biological processes, for example the initiation of downstream cellular pathways through selective receptor–ligand interactions.^6–9^ They can also transport endogenous and exogenous ligand molecules from the plasma to the intracellular milieu (for example to the lysosome for degradation),^10,11^ maintaining the balance of these species in the blood stream. However, in some cases Receptor Mediated Entries (RMEs) are also implicated in the invasion of viruses^12–15^ and progression and metastasis of cancers,^16–20^ thereby highlighting the importance of probing RMEs processes for disease diagnosis and therapy.

**Fig. 1:**
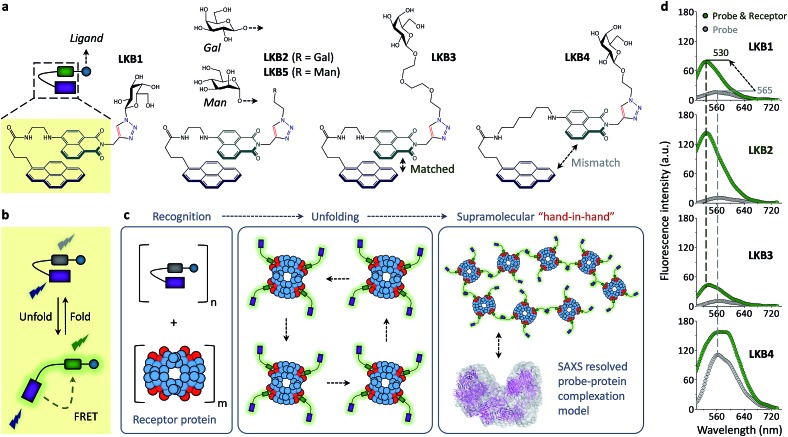
Structures of the foldable glycoprobes synthesized and schematic illustration of their ability to crosslink receptor proteins. (a) Structure of the folded glycoprobes **LKB1–LKB5**. Schematic illustration of (b) the folded and unfolded states of glycoprobe and (c) supramolecular assembly of the glycoprobe with a protein receptor. (d) Fluorescence spectra of **LKBs** (5 μM in 0.05 M PBS, pH 7.4; excitation wavelength: 345 nm) in the absence and presence of a receptor protein, peanut agglutinin (PNA, 25 μM).

Here we report a single-molecular glycoprobe capable of simultaneously detecting and crosslinking receptor proteins. Through the coupling of a glycoligand to a bis-fluorophore conjugate we have produced a foldable glycoprobe with minimal fluorescence in an aqueous medium (Fig. 1a). Subsequently, association of the probe with a selective receptor protein rapidly enhances the fluorescence by unfolding the probe (Fig. 1b), and this releases an additional fluorophore “hand” which can facilitate cross-linking of the proteins to form unique supramolecular assemblies, as determined by small-angle X-ray scattering (SAXS) (Fig. 1c). Furthermore, the foldable glycoprobe has proven effective in tracking the endocytic cycle of a transmembrane receptor protein.

## Results and discussion

The folded glycoprobes were synthesized by introducing a glycoligand (galactose that can be recognized by galactose-selective receptor proteins) to a bis-fluorophore conjugate using a click reaction.^21,22^ To synthesize the conjugate, a naphthalimide was grafted to 1-pyrenebutyrate *via* a flexible alkyl chain to facilitate intramolecular interaction of the two fluorophores (Fig. 1, Scheme S1†).^23–25^ Therefore, we prepared **LKB1**, **LKB2**, **LKB3** and **LKB4** with increasing linker chain length between the glycoligand and the conjugate, and **LKB5** using a mannose instead of galactose (Fig. 1 and Scheme S2†).

With the glycoprobes in hand, we measured their fluorescence in the absence and presence of a galactose-selective lectin, peanut agglutinin (PNA), in an aqueous buffer solution (Tris–HCl, pH 7.4). We determined that **LKB1**, **LKB2** and **LKB3** displayed minimal fluorescence in the buffer medium with excitation at 345 nm, whereas **LKB4** had strong fluorescence with an emission maxima at *ca.* 565 nm (Fig. 1d). Addition of PNA caused a large fluorescence enhancement of **LKB1–LKB3** with a blue-shifted emission maxima (*ca.* 530 nm), but only slight fluorescence enhancement with **LKB4** (Fig. 1d). Among the three probes with weak initial fluorescence, **LKB2** displayed the largest fluorescence enhancement in the presence of PNA. This suggests that the association of **LKB2** with PNA is optimal. In contrast, an increase of linker chain length between the two fluorophores led to an increased initial fluorescence of **LKB4**, probably suggesting sub-optimal matching between the naphthalimide and pyrene.

**LKB2** with the best PNA sensitivity was selected to investigate the mechanism by which the fluorescence of the probe is enhanced (Fig. S1†). We first determined that the absorbance (A) band of **LKB2** red-shifted with respect to the unconjugated naphthalimide (**N**) and pyrene (**P**) (Scheme S1†) (Fig. S1a and Scheme S1†). This suggests an intimacy between the two fluorophores.^26,27^ Shown in Fig. S1b† are the stacked absorbance (A) and emission (E) spectra of **P** and **N**, implying a possibility of Förster resonance energy transfer (FRET) from **P** to **N** because of good overlap between the emission band of the former with the absorbance band of the latter. Using these observations we deduce that the two hydrophobic fluorophores are stacked in aqueous solution to adopt an excimer-like, folded conformation with red-shifted (with respect to naphthalimide) and weak fluorescence emission.^28,29^ Subsequent unfolding of the probe through association with a protein produces a typical naphthalimide emission^30^ through FRET, upon excitation of pyrene (345 nm) (Fig. 1b). This deduction was corroborated by two additional assays. Firstly, we determined that an increase of temperature (*T*) caused the emission peak of the probe to gradually blue-shift (Fig. S1c†). A similar blue-shift was observed for the probe in a range of organic solvents (Org) when compared to that in full aqueous buffer (Aq) (Fig. S1d†). Both assays imply that the folded structure of the probe could be extended and opened by tuning of the solvent conditions.

Next, we determined that the fluorescence enhancement of **LKB2** and the mannose-appended **LKB5** was dependent on the concentration of PNA (Fig. S1e†) and a mannose-selective lectin, concanavalin A (Con A) (Fig. S1f†), respectively. In addition, the fluorescence enhancement was observed to be specific for selective lectin receptors (**LKB2** for PNA and **LKB5** for Con A and another mannose-selective lectin, lens culimaris agglutinin [LcA]) over a panel of unselective lectins and proteins including wheat germ agglutinin, pisum sativum lectin, bovine serum albumin, pepsin, lysin, human cytochrome c and ribonuclease A (Fig. S1g and h†).

**Fig. 2:**
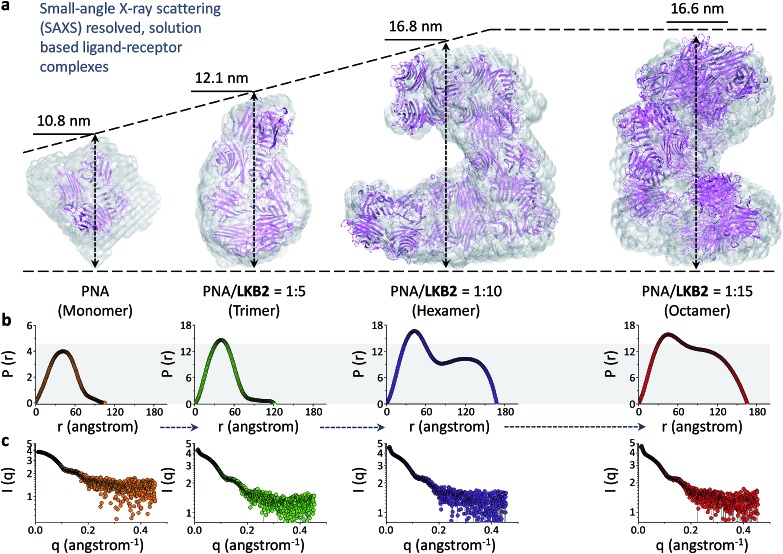
SAXS of the assembly between folded glycoprobe and PNA (peanut agglutinin) (PDB 2PEL). (a) Atomic model of PNA alone and with increasing **LKB2** displayed as C_α_ chain and superimposed to the *ab initio* low-resolution models of PNA obtained by DAMAVER (semi-transparent beads). (b) Interatomic distance distribution function, *P*(*r*), of the X-ray scattering patterns of different **LKB2**/PNA (w/w) ensembles. (c) X-ray scattering patterns of different **LKB2**/PNA ensembles.

With the biospecificity of the glycoprobes determined, we sought to determine the mode of association between the probe and PNA using SAXS, a technique that can provide detailed structural information for macromolecules.^31,32^ On the basis of the single merged X-ray scattering profile obtained from PNA solutions with or without **LKB2**, *ab initio* low-resolution models were calculated (Fig. 2c). The results suggest a gradual aggregation of PNA in the presence of increasing concentrations of the glycoprobe, forming an octamer with a final probe/protein ratio of 1/15 (w/w) (Fig. 2a). Fig. 2b shows the interatomic distance distribution function, *P*(*r*), which similarly indicates an increase in maximum diameter (*D*_max_) of the protein with increasing probe concentration. Recently, a solid-state complex structure formed between a glucosyl rhodamine and Con A has been resolved by X-ray crystallography,^33^ revealing that while the glucosyl moiety docks into the carbohydrate recognition domain of Con A, the rhodamine tail can dimerize and facilitate aggregation of the proteins. With this X-ray crystal data and our SAXS results we suggest that association of the glycoprobe with PNA can unfold the bis-fluorophore conjugate, releasing the pyrene tail. These released pyrene tails can then stack with each other to crosslink the proteins, forming protein aggregates as determined using SAXS (Fig. 1c).

**Fig. 3:**
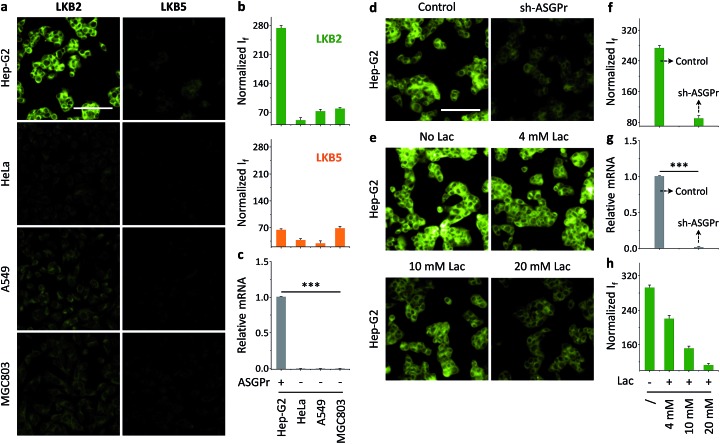
Receptor-targeting cell imaging of foldable glycoprobes. Fluorescence imaging (a) and quantification (b) of **LKB2** (20 μM) and **LKB5** (20 μM) for different human cancer cell lines (Hep-G2 = human liver cancer; HeLa = human cervical cancer; A549 = human lung cancer; MGC803 = human gastric cancer). (c) Relative mRNA level of different cancer cells determined by real-time quantitative polymerase chain reaction (RT-qPCR) (***P < 0.001). Fluorescence imaging (d) and quantification (f) of **LKB2** (20 μM) for Hep-G2 with (sh-ASGPr) or without (control) knockdown of ASGPr (asialoglycoprotein receptor). (g) Relative mRNA level of sh-ASGPr and control determined by RT-qPCR (***P < 0.001). Fluorescence imaging (e) and quantification (h) of **LKB2** (20 μM) for Hep-G2 cells preincubated with increasing lactose (Lac). For all fluorescence images, the excitation wavelength was 345 nm and emission channel 450–550 nm (scale bar: 100 μm, which is applicable to all images).

Having established the solution-based glycoprobe-protein complexation, we investigated the interaction of **LKB2** with a transmembrane receptor at the cellular level. We used a human liver cancer cell line that expresses a galactose-selective asialoglycoprotein receptor (ASGPr)^34–36^ and other cell lines including HeLa (human cervix cancer), A549 (human lung cancer) and MGC803 (human gastric cancer) as controls. We observed that incubation of **LKB2** with the cells resulted in a strong fluorescence for Hep-G2, but not in the control cells (Fig. 3a and b). This is in agreement with the ASGPr expression level of the cell lines determined by real-time quantitative polymerase chain reaction (Fig. 3c). Meanwhile, treatment of the cells with **LKB5** bearing a mannose precursor did not produce fluorescence in all the cell lines used (Fig. 3a and b).

We also used a modified Hep-G2 cell line (sh-ASGPr) with a suppressed ASGPr expression by sh-RNA plasmid transfection^37^ with **LKB2**. The result suggested that knockdown of ASPGr significantly reduces the fluorescence imaging effect of the glycoprobe (Fig. 3d and f), and the fluorescence intensity produced by **LKB2** in sh-ASGPr and control (Hep-G2 transfected with a scrambled sh-RNA) is consistent with the receptor expression level of the two cell lines (Fig. 3g). A competition assay by pre-treatment of free lactose with Hep-G2 showed that the fluorescence of **LKB2** in Hep-G2 could be gradually reduced with increasing lactose concentrations (Fig. 3e and h). Meanwhile, a cell viability assay suggested that both **LKB2** and **LKB5** were not toxic to Hep-G2 and sh-ASGPr at a concentration five-fold higher than that used for cell imaging (Fig. S2†). These data suggest that the glycoprobe can be used to target a transmembrane receptor for cell imaging experiments.

**Fig. 4:**
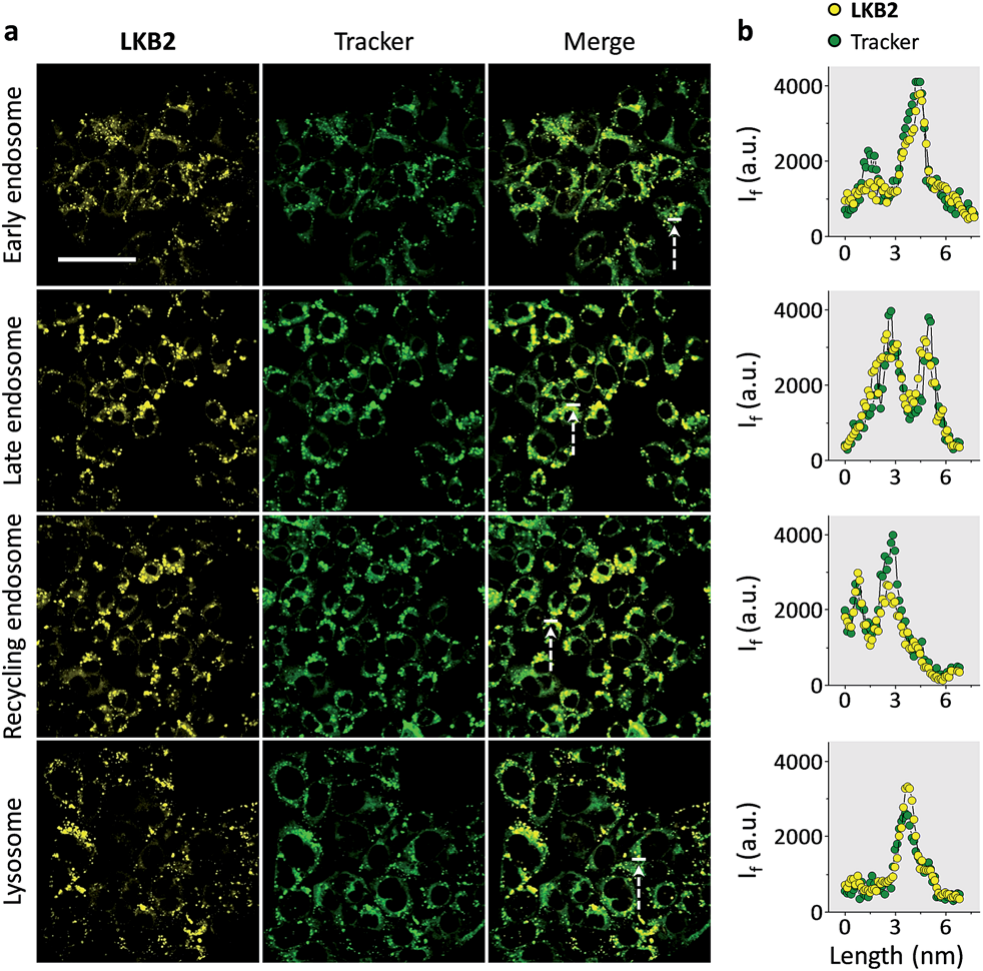
Tracking the endocytic cycle of a transmembrane receptor by foldable glycoprobes. Fluorescence imaging (a) and quantitative co-localization (b) (the line-cross sections shown in the merged images were used for quantification) of **LKB2** (80 μM) and green fluorescence protein (GFP)-tagged antibody trackers of different subcellular compartments using confocal scanning laser microscopy (excitation: 400 nm; emission channel: 450–550 nm; scale bar: 40 μm, which is applicable to all images).

Finally, we tested the ability of **LKB2** to track the endocytosis of ASGPr by confocal laser scanning microscopy. Typical endocytic pathways for ASGPr have been suggested, which depend on the formation of early endosomes after ligand–receptor binding and a segregation of ligand and receptor, where the ligand is delivered by late endosomes to the lysosome for degradation and receptor recycled to cell surface by recycling endosomes.^10^ As a result, we used Hep-G2 cells transfected with different GFP (green fluorescence protein)-tagged antibody trackers of the subcellular compartments to monitor the glycoprobe internalization. We observed strong fluorescence signals in all the compartments tracked (Fig. 4a); the fluorescence of **LKB2** co-localized well with those of GFP trackers (Fig. 4b). We also used a control naphthalimide galactoprobe (**LKB6**) without the pyrene tail for cell imaging (Fig. S3†). The results indicated that the control compound had a much weaker Hep-G2 imaging effect than **LKB2**, probably suggesting the importance of the foldable motif of the latter to underpin the receptor binding. Although more evidence is needed to elucidate the dynamic and complex receptor-probe binding processes in cells, the confocal imaging results obtained here highlight the effectiveness of the foldable probe to track the intracellular localizations of receptor proteins.

## Conclusions

With this research we have developed a foldable glycoprobe that can be used to simultaneously detect and modulate the construction of protein receptors. While the probe displayed specific fluorescence enhancements in the presence of a selective receptor in aqueous buffer solution, results obtained from SAXS suggest a concentration-dependent crosslinking of proteins by the probe. The unique probe-protein oligomeric complexation determined represents an exceptional example of protein aggregation by small-molecular probes. The observation that the glycoprobe has proven amenable to tracking the endocytic cycle of a transmembrane receptor highlights its potential for use in elucidating the intracellular localization of functional receptor proteins. This research also offers a new insight into the construction of supramolecular constructs based on the bio-recognition between a foldable small-molecular probe and biomacromolecules.
