# Supplementary material for: Foldable glycoprobes capable of fluorogenic crosslinking of biomacromolecules
Source: Chem Sci. 2016 Jul 12;7(10):6325–9. doi: 10.1039/c6sc02366e (PMC5450440; doi:10.1039/c6sc02366e)

This journal is © The Royal Society of Chemistry 2016

Supplementary information for:

Foldable glycoprobe capable of fluorogenic crosslinking of biomacromolecules

Kai-Bin Li,[1] Na Li,[3] Yi Zang,[2] Guo-Rong Chen,[1] Jia Li,[2,*] Tony D James,[4] Xiao-Peng He[1,*] & He Tian[1]

[1]*Key Laboratory for Advanced Materials & Institute of Fine Chemicals, School of Chemistry and Molecular Engineering, East China University of Science and Technology, Shanghai 200237, China*
[2]*National Center for Drug Screening, State Key Laboratory of Drug Research, Shanghai Institute of Materia Medica (SIMM), Chinese Academy of Sciences (CAS), 189 Guo Shoujing Rd., Shanghai 201203, PR China*
[3]*National Center for Protein Science Shanghai, Shanghai Institutes of Biological Sciences, Chinese Academy of Sciences, Shanghai 200031, China*
[4]*Department of Chemistry, University of Bath, Bath, BA2 7AY, UK*

Corresponding authors:
jli@simm.ac.cn (J. Li)
xphe@ecust.edu.cn (X.-P. He)

Contents list

S1. Figures S1-S3

S2. Experimental section

S3. Additional references

S4. Original spectral copies

S1. Figures S1-S3

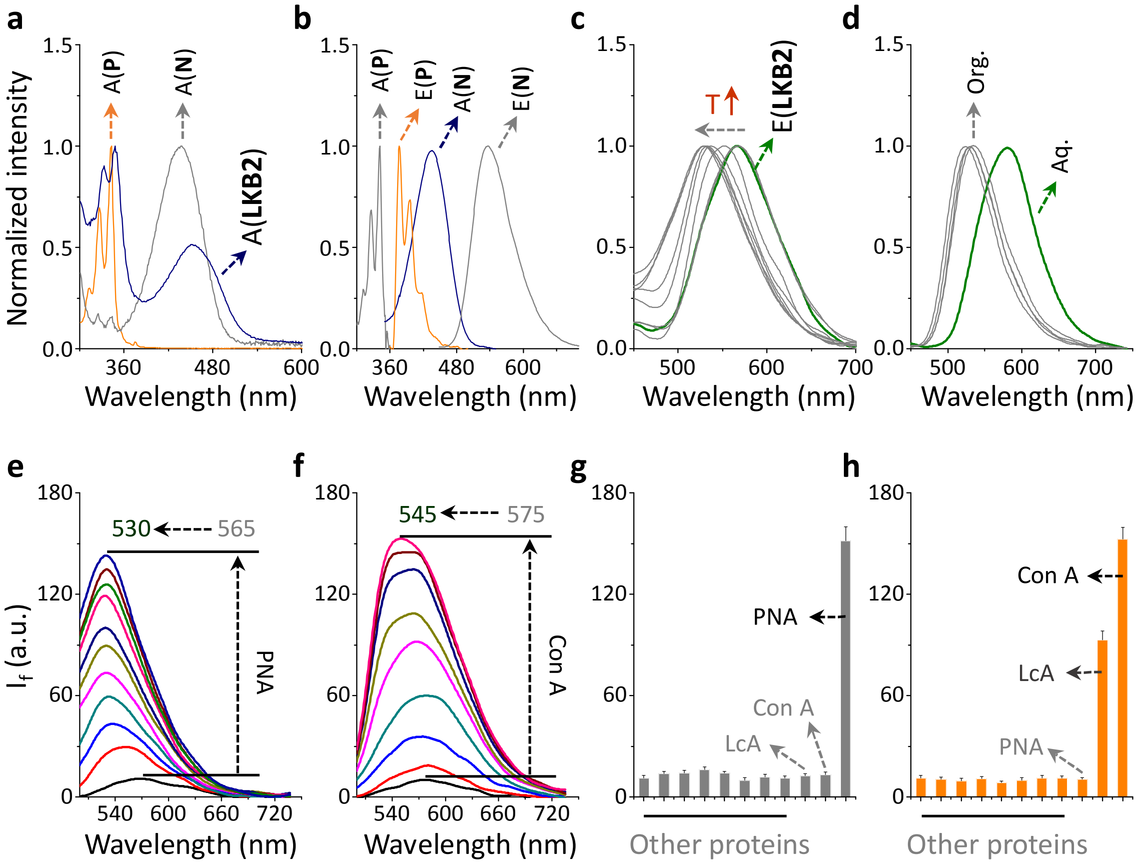

Figure S1. (a) Stacked UV-vis absorbance spectra of unconjugated pyrene (**P**), naphthalimide (**N**) and **LKB2** (structures are shown in Scheme S1). (b) Stacked UV-vis absorbance and fluorescence emission spectra of **P** and **N**. (c) Stacked fluorescence spectra of **LKB2** with increasing temperature (right curve to left: 10 to 90 °C; interval: 10 °C). (d) Stacked fluorescence spectra of **LKB2** in organic (DMSO, MeCN, EtOH, MeOH) and aqueous (0.05 M, PBS, pH 7.4) solutions. Fluorescence titration of (e) **LKB2** with increasing peanut agglutinin (PNA, 0-25 µM) and (f) **LKB5** with increasing concanavalin A (Con A, 0-25 µM). Fluorescence change of (g) **LKB2** and (h) **LKB5** in the presence of 25 µM of different proteins (other proteins from left to right: wheat germ agglutinin, Pisum sativum lectin, bovine serum albumin, pepsin, lysin, human cytochrome c and ribonuclease A). Unless otherwise stated, all fluorescence spectra were measured with a compound concentration of 5 µM in 0.05 M PBS (pH 7.4) and an excitation wavelength of 345 nm.

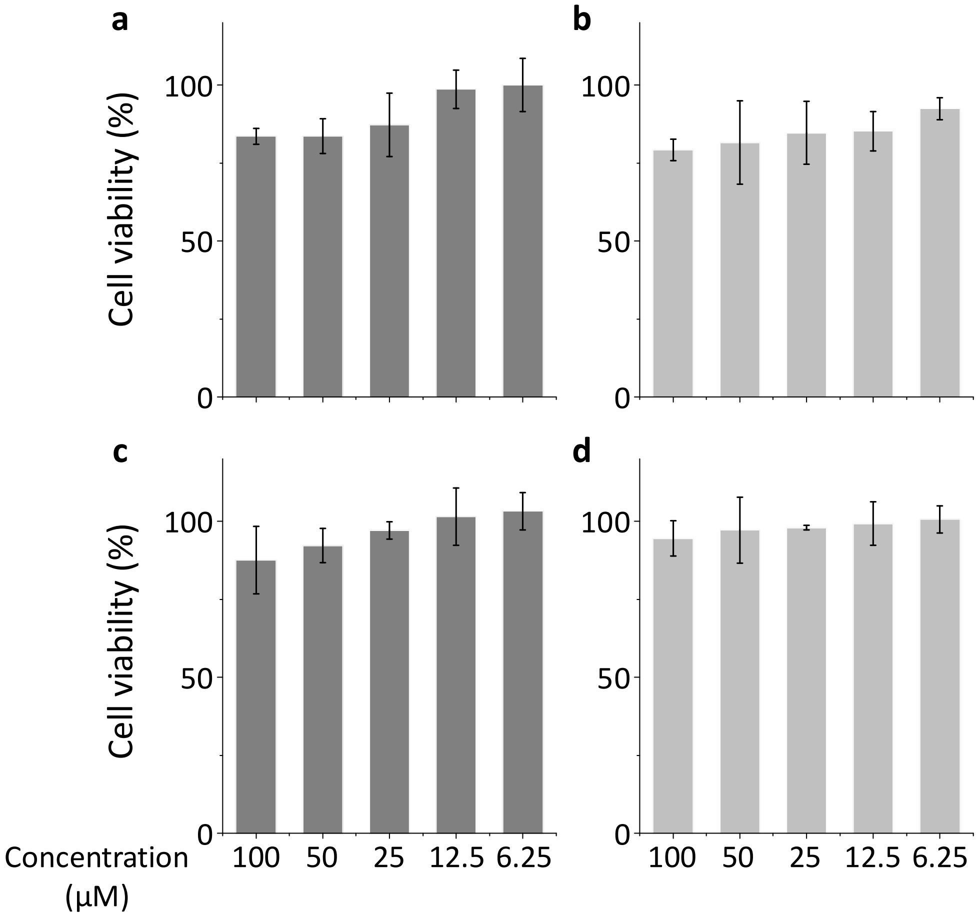

Figure S2. Viability of (a, c) sh-ASGPr and (b, d) Hep-G2 in the presence of increasing (a, b) **LKB2** and (c, d) **LKB5** determined by an MTS assay.

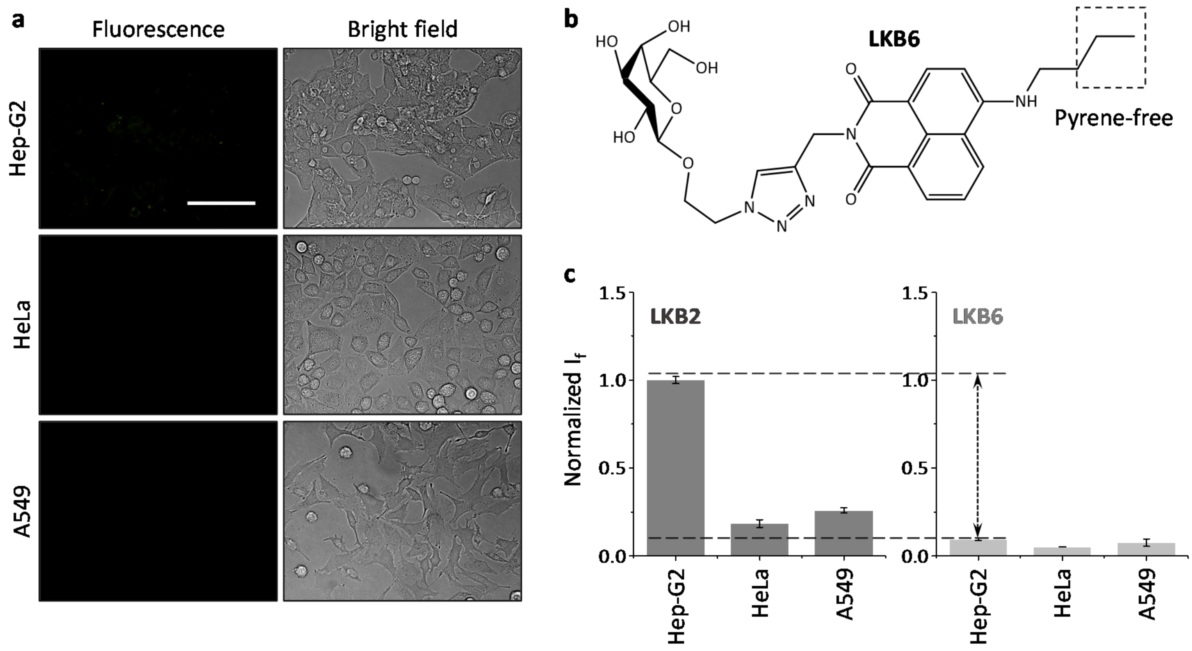

Figure S3. (a) Fluorescence imaging using **LKB6** (20 μM) for different human cancer cell lines (Hep-G2 = human liver cancer; HeLa = human cervical cancer; A549 = human lung cancer). (b) Structure of the pyrene-tail-free **LKB6** used as control for cell imaging. (c) Fluorescence quantification of **LKB2** (20 μM) and **LKB6** (20 μM) for different cells. For all fluorescence images, the excitation wavelength was 345 nm and emission channel 450-550 nm (scale bar: 100 μm, which is applicable to all images).

S2. Experimental section

S2.1. General remarks

All purchased chemicals and reagents are of analytical grade. Solvents were purified by standard procedures. Reactions were monitored by TLC (thin-layer chromatography) using E-Merck aluminum precoated plates of Silica Gel. ^{1}H and ^{13}C NMR spectra were recorded on a Bruker AM-400 spectrometer using tetramethylsilane (TMS) as the internal standard (chemical shifts in parts per million). High resolution mass spectra (HRMS) were recorded on a Waters LCT Premier XE spectrometer using standard conditions (ESI, 70 eV). High performance liquid chromatography (HPLC) was carried out on an Agilent 1100 Series equipment. Proteins were purchased from Sigma-Aldrich.

S2.2. Synthesis of foldable glycoprobes

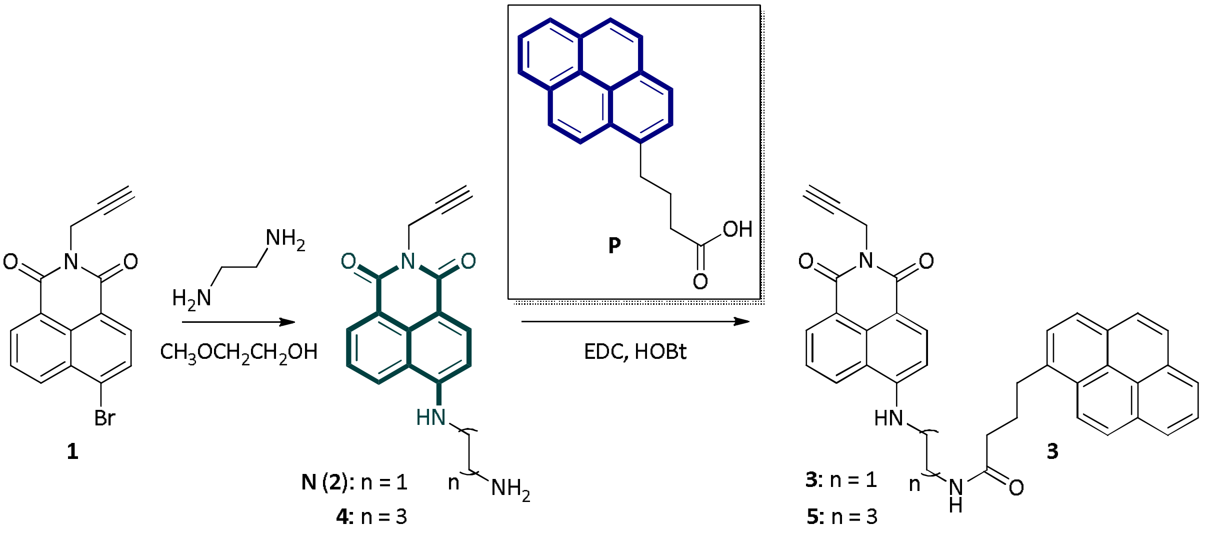

Scheme S1. Synthesis of intermediates **2-5**.

Synthesis of N (2). Compound **1** (1.00 g, 3.20 mmol)[1] and ethanediamine (0.38 g, 6.40 mmol) were dissolved in 2-methoxyethanol (20 mL), and the mixture was heated to reflux overnight. The solvent was evaporated under reduced pressure and the residue was purified by flash column chromatography with CH_2Cl_2/MeOH (95:5, *v/v*) to produce **2** (**N**) as a yellow crystal (0.94 g, 95 %). ^{1}H NMR (400 MHz, CD_3OD): δ 2.53 (t, J = 1.6 Hz, 1H), 3.06 (t, J = 6.0 Hz, 2H), 3.37 (t, J = 12.0 Hz, 2H), 3.58 (t, J = 6.4 Hz, 2H), 4.88 (s, 2H), 6.88 (d, J = 8.0 Hz, 1H), 7.69 (t, J = 8.0 Hz, 1H), 8.41 (d, J = 8.0 Hz, 1H), 8.55-8.60 (m, 2H); ^{13}C NMR (400 MHz, $CDCl_3$) δ 28.6, 39.7, 45.8, 72.4, 79.9, 103.9, 107.0, 120.1, 121.3, 124.2, 129.0, 129.3, 130.9, 134.4, 151.1, 161.9, 163.0. HR-ESI-MS *m*/*z*: $[M + H]^+$ calcd. for 294.1243, found 294.1235.

*Synthesis of **3***. To a solution of **2** (500 mg, 1.70 mmol) and pyrenebutyric acid (**P**) (500 mg, 1.73 mmol) in CH_2Cl_2 were added EDC·HCl (1 g, 5.21 mmol) and HOBt (230 mg, 1.70 mmol), and the mixture was stirred over night at room temperature. The resulting mixture was then diluted with CH_2Cl_2 and water, washed successively with water and brine, and extracted with CH_2Cl_2. The combined organic layer was dried over $MgSO_4$, filtered and concentrated in vacuum. The resulting residue was purified by column chromatography on silica gel (CH_2Cl_2/MeOH = 50:1, *v/v*) to afford compound **3** as a yellow crystal (900 mg, 94%). ^{1}H NMR (400 MHz, DMSO-d_6): δ 2.02 (m, 4H), 2.28 (t, J = 2.8 Hz, 2H), 3.08 (s, 1H), 3.24 (t, J = 8.0 Hz, 2H), 3.46 (m, 4H), 4.68 (s, 2H), 6.87 (d, J = 8.8 Hz, 1H), 7.19-7.42(m, 2H), 7.62 (t, J = 8.0 Hz, 1H), 7.86 (d, J = 8.0 Hz, 1H), 7.92 (t, J = 4.0 Hz, 1H), 8.04 (t, J = 8.0 Hz, 1H), 8.11-8.29 (m, 5H), 8.34 (d, J = 4.0 Hz, 1H), 8.59 (d, J = 4.0 Hz, 1H); ^{13}C NMR (100 MHz, DMSO-d_6): δ 27.8, 29.1, 32.6, 35.5, 37.9, 43.5, 72.9, 80.4, 104,3, 107.7, 120.5, 121.8, 123.8, 124.5, 124.6, 124.8, 125.2, 125.3, 125.4, 126.5, 126.9, 127.5, 127.8, 127.9, 128.5, 129.1, 129.7, 129.8, 130.8, 131.3, 134.9, 136.8, 151.4, 162.4, 163.4, 173.5. HR-ESI-MS *m/z*: $[M + H]^+$ calcd. for 564.2287, found 564.2285.

*Synthesis of **4***. Compound **1** (300 mg, 0.96 mmol) and hexamethylenediamine (557 mg, 4.80 mmol) were dissolved in 2-methoxyethanol (20 mL), and the mixture was heated to reflux overnight. Then, solvent was evaporated under reduced pressure and the residue was purified by column chromatography with CH_2Cl_2/MeOH (95:5, *v/v*) to produce **4** as a yellow crystal (318 mg, 95 %). ^{1}H NMR (400 MHz, CD_3OD): δ 1.31-1.56 (m, 8H), 1.79-1.86 (m, 2H), 2.62-2.69 (m, 2H), 3.45 (t, J = 7.2 Hz, 2H), 4.85 (s, 2H), 6.73 (d, J = 8.8Hz, 1H), 7.60 (t, J = 8.0 Hz, 1H), 8.31 (d, J = 8.8 Hz, 1H), 8.46-8.49 (m, 2H); ^{13}C NMR (100 MHz, CD_3OD): δ 25.8, 26.2, 27.7, 27.8, 28.6, 29.2, 42.7, 72.4, 79.9, 103.8, 106.8, 120.1, 121.3, 124.2, 129.1, 129.4, 130.9, 134.5, 151.0, 162.0, 163.0. HR-ESI-MS *m/z*: $[M + H]^+$ calcd. for 350.1869, found 350.1833.

*Synthesis of **5***. To a solution of **4** (593 mg, 1.70 mmol) and **P** (500 mg, 1.73 mmol) in CH_2Cl_2, were added EDC·HCl (1 g, 5.21 mmol) and HOBt (230 mg, 1.70 mmol), and the mixture was stirred over night at room temperature. The resulting mixture was then diluted with CH_2Cl_2 and water, washed successively with water and brine, and extracted with CH_2Cl_2. The combined organic layer was dried over $MgSO_4$, filtered and concentrated in vacuum. The resulting residue was purified by column chromatography on silica gel (CH_2Cl_2/MeOH = 50:1, *v/v*) to afford compound **5** as a yellow crystal (947 mg, 90%). ^{1}H NMR (400 MHz, DMSO-d_6): δ 1.23-1.43 (m, 8H), 1.70 (m, 2H), 1.97-2.04 (m, 2H), 2.19-2.24 (m, 3H), 3.03-3.10 (m, 3H), 3.29 (t, J = 8.0 Hz, 2H), 4.73 (s, 2H), 6.72 (d, J = 8.0 Hz, 1H), 7.64 (t, J = 8.0 Hz, 1H), 7.79-7.86 (m, 2H), 7.91 (d, J = 8.0 Hz, 1H), 8.01-8.06 (m, 1H), 8.10-8.27 (m, 5H), 8.33-8.37 (m, 1H), 8.41

(d, J = 7.2 Hz, 1H), 8.68 (d, J = 8.0 Hz, 1H). HR-ESI-MS m/z: $[M + H]^+$ calcd. for 620.2913, found 620.2911.

*General procedure for the synthesis of **LKB1-5***. To a solution of sugar azide (**6-9**)[2-4] (1 equiv.) and alkyne (**3** or **5**) (1 equiv.) in CH_2Cl_2/H_2O (5 mL/5 mL) were added sodium ascorbate (6 equiv.) and $CuSO_4 \cdot 5H_2O$ (4 equiv.). The resulting mixture was stirred overnight at room temperature. The resulting mixture was then diluted with CH_2Cl_2 and water, washed successively with water and brine, and extracted with CH_2Cl_2. The combined organic layer was dried over $MgSO_4$, filtered and concentrated in vacuum. The resulting residue was directly dissolved in $MeOH/H_2O/Et_3N$ (8:1:1, *v/v/v*), and the mixture was stirred overnight at room temperature. Solvent was then removed in vacuum and the residue directly purified by column chromatography on silica gel to afford the final products.

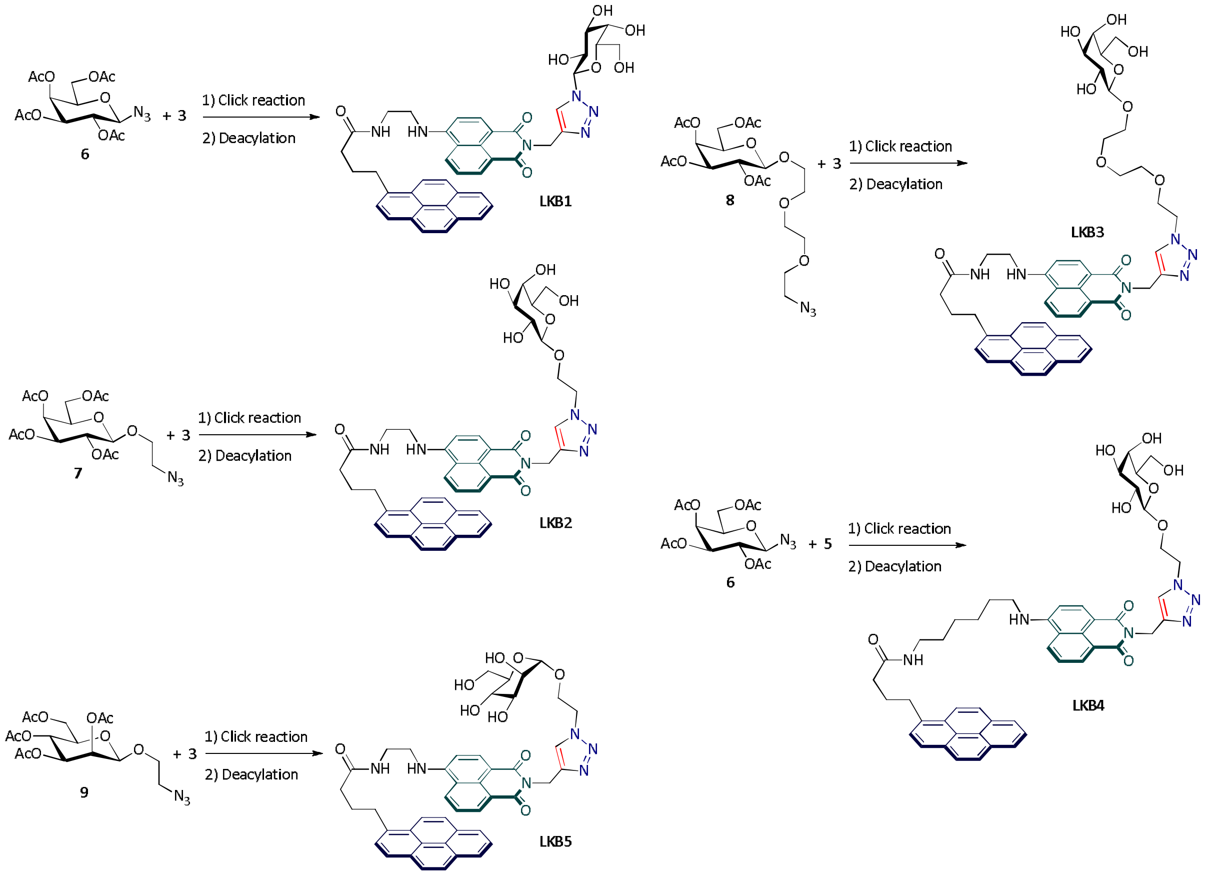

Scheme S2. Synthesis of glycoprobes **LKB1-5**.

*Synthesis of **LKB1***. From alkyne **3** (300 mg, 0.53 mmol) and azide **6** (240 mg, 0.64 mmol), column chromatography (CH_2Cl_2/MeOH = 10:1 to 5:1, *v/v*) afforded **LKB1** as a yellow solid (338 mg, 83.1%). ^1H NMR (400 MHz, DMSO-d_6) δ 2.03 (t, J = 8.0 Hz, 2H), 2.29 (t, J = 7.2

Hz, 2H), 3.26 (t, J = 8.0 Hz, 2H), 3.48-3.52 (m, 8H), 3.64-3.70 (m, 2H), 4.56 (d, J = 4.4 Hz, 1H), 4.68 (t, J = 6.0 Hz, 1H), 4.90 (d, J = 5.2 Hz, 1H), 5.18 (d, J = 6.0 Hz, 1H), 5.23 (s, 2H), 5.33 (d, J = 5.2 Hz, 1H), 6.87 (d, J = 8.4 Hz, 1H), 7.60-7.65 (m, 1H), 7.85-7.94 (m, 2H), 8.03-8.36 (m, 10H), 8.59 (d, J = 8.4 Hz, 1H); ^{13}C NMR (100 MHz, DMSO-d_6): δ 27.8, 32.6, 35.5, 43.5, 60.9, 68.6, 69.7, 70.7, 73.7, 74.1, 78.0, 78.9, 88.5, 91.1, 104.2, 108.1, 120.6, 122.2, 122.3, 123.8, 124.5, 124.6, 124.8, 125.2, 125.3, 125.4, 126.6, 127.0, 127.6, 127.9, 128.0, 128.5, 128.9, 129.7, 130.0, 130.8, 131.2, 131.3, 134.8, 136.8, 151.2, 163.1, 173.5. HR-ESI-MS *m/z*: $[M + H]^+$ calcd. for 769.2986, found 769.2984. HPLC: t_R = 4.7 min over 15 min of eluent (90% CH_3OH and 10% H_2O), purity 96.5%.

*Synthesis of **LKB2***. From alkyne **3** (100 mg, 0.18 mmol) and azide **7** (90 mg, 0.21 mmol), column chromatography (CH_2Cl_2/MeOH = 10:1 to 5:1, *v/v*) afforded **LKB2** as a yellow solid (119 mg, 81.7%). ^1H NMR (400 MHz, DMSO-d_6) δ 2.02 (t, J = 8.0 Hz, 2H), 2.28 (t, J = 7.2 Hz, 2H), 3.22-3.30 (m, 4H), 3.34-3.73 (m, 8H), 3.81-4.06 (m, 2H), 4.14 (d, J = 6.8 Hz, 1H), 4.36 (d, J = 4.4 Hz, 1H), 4.48 (t, J = 4.8 Hz, 2H), 4.56 (d, J = 6.0 Hz, 1H), 4.71 (d, J = 4.4 Hz, 1H), 4.87 (d, J = 3.6 Hz, 1H), 5.21 (s, 2H), 6.85 (d, J = 8.4 Hz, 1H), 7.61 (t, J = 8.0 Hz, 1H), 7.87 (d, J = 7.6 Hz, 2H), 8.02-8.30 (m, 9H), 8.34 (d, J = 7.2 Hz, 1H), 8.57 (d, J = 8.4 Hz, 1H); ^{13}C NMR (100 MHz, DMSO-d_6): δ 27.3, 32.1, 35.0, 42.9, 60.4, 69.1, 70.3, 73.3, 75.4, 76.3, 78.9, 79.1, 82.3, 83.4, 99.5, 103.5, 103.7, 107.7, 108.1, 114.3, 114.7, 120.0, 123.3, 124.0, 124.1, 124.3, 124,4, 124.7, 124.8, 124.9, 126.1, 126.4, 127.1, 127.3, 127.4, 129.2, 130.3, 130.7, 130.8, 134.3, 134.4, 136.3, 162.5, 163.4, 173.0. HR-ESI-MS *m/z*: $[M + H]^+$ calcd. for 813.3248, found 813.3246. HPLC: t_R = 6.0 min over 15 min of eluent (90% CH_3OH and 10% H_2O), purity 97.0%.

*Synthesis of **LKB3***. From alkyne **3** (90 mg, 0.16 mmol) and azide **8** (58 mg, 0.17 mmol), column chromatography (CH_2Cl_2/MeOH = 10:1 to 5:1, *v/v*) afforded **LKB3** as a yellow solid (118 mg, 82.1%). ^1H NMR (400 MHz, DMSO-d_6) δ 2.02 (t, J = 8.0 Hz, 2H), 2.28 (t, J = 7.2 Hz, 2H), 3.23-3.25 (m, 4H), 3.42-3.62 (m, 11H), 3.75 (t, J = 5.2 Hz, 2H), 4.07 (d, J = 7.2 Hz, 1H), 4.35 (d, J = 4.4 Hz, 1H), 4.43 (t, J = 5.2 Hz, 2H), 4.56 (t, J = 5.6 Hz, 1H), 4.69 (d, J = 4.4 Hz, 1H), 4.83 (d, J = 4.0 Hz, 1H), 5.21 (s, 2H), 6.87 (d, J = 8.4 Hz, 1H), 7.62 (t, J = 8.0 Hz, 1H), 7.86-7.91 (m, 2H), 8.04 (t, J = 7.6 Hz, 1H), 8.11-8.30 (m, 8H), 8.34 (d, J = 7.2 Hz, 1H), 8.58 (d, J = 8.0 Hz, 1H). HR-ESI-MS *m/z*: $[M + H]^+$ calcd. for 901.3772, found 901.3766. HPLC: t_R = 6.8 min over 15 min of eluent (90% CH_3OH and 10% H_2O), purity 96.7%.

*Synthesis of **LKB4***. From alkyne **5** (300 mg, 0.48 mmol) and azide **6** (217 mg, 0.58 mmol), column chromatography (CH_2Cl_2/MeOH = 10:1 to 5:1, *v/v*) afforded **LKB4** as a yellow solid (341 mg, 86.0%). ^{1}H NMR (400 MHz, DMSO-d_6) δ 1.27-1.44 (m, 8H), 1.70 (t, J = 6.0 Hz, 2H), 2.01 (t, J = 7.2 Hz, 2H), 2.22 (t, J = 7.2 Hz, 2H), 3.05-3.10 (m, 2H), 3.32 (t, J = 7.2 Hz, 2H), 3.43-3.52 (m, 4H), 3.64-3.71 (m, 2H), 4.57 (d, J = 4.2 Hz, 1H), 4.71 (t, J = 6.0 Hz, 1H), 4.97 (d, J = 5.6 Hz, 1H), 5.20 (d, J = 6.0 Hz, 1H), 5.27 (s, 2H), 5.43 (d, J = 9.2 Hz, 1H), 6.74 (d, J = 8.4 Hz, 1H), 7.65 (t, J = 8.0 Hz, 1H), 7.79-7.93 (m, 3H), 8.01-8.25 (m, 7H), 8.35 (d, J = 9.2 Hz, 1H), 8.42 (d, J = 7.2 Hz, 1H), 8.69 (d, J = 8.4 Hz, 1H); ^{13}C NMR (100 MHz, DMSO-d_6): δ 26.2, 26.3, 27.6, 29.1, 32.2, 35.0, 38.3, 42.7, 60.4, 68.5, 69.2, 69.5, 73.6, 78.4, 88.0, 89.5, 101.9, 103.7, 106.3, 107.2, 120.1, 127.1, 123.4, 124.1, 124.2, 124.7, 124.9, 126.1, 126.4, 127.1, 127.3, 127.3, 127.5, 128.1, 129.2, 129.5, 129.6, 130.3, 130.8, 131.2, 134.4, 134.5, 136.5, 150.8, 162.6, 163.6, 171.7. HR-ESI-MS *m/z*: [M + H]$^+$ calcd. for 825.3612, found 825.3599. HPLC: t_R = 4.6 min over 15 min of eluent (90% CH_3OH and 10% H_2O), purity 96.8%.

*Synthesis of **LKB5***. From alkyne **3** (204 mg, 0.36 mmol) and azide **9** (181 mg, 0.43 mmol), column chromatography (CH_2Cl_2/MeOH = 10:1 to 5:1, *v/v*) afforded **LKB5** as a yellow solid (228 mg, 78.1%). ^{1}H NMR (400 MHz, DMSO-d_6) δ 2.02 (t, J = 8.0 Hz, 2H), 2.28 (t, J = 6.4 Hz, 2H), 3.15-3.27 (m, 4H), 3.41-3.65 (m, 8H), 3.67-3.95 (m, 2H), 4.28-4.60 (m, 5H), 4.68 (d, J = 4.4 Hz, 1H), 4.71 (d, J = 4.4 Hz, 1H), 5.21 (s, 2H), 6.86 (d, J = 8.8 Hz, 1H), 7.61 (t, J = 8.0 Hz, 1H), 7.86-7.93 (m, 3H), 8.01-8.31 (m, 8H), 8.34 (d, J = 6.8 Hz, 1H), 8.58 (d, J = 8.8 Hz, 1H); ^{13}C NMR (100 MHz, DMSO-d_6): δ 27.3, 32.1, 35.0, 37.4, 42.9, 46.6, 49.1, 61.1, 64.7, 64.8, 66.7, 70.0, 70.8, 74.1, 99.7, 103.7, 107.6, 120.0, 121.7, 123.3, 123.4, 123.5, 124.0, 124.1, 124.3, 124.4, 124.7, 124.8, 124.9, 125.3, 126.1, 126.4, 127.1, 127.3, 127.4, 128.0, 128.4, 129.2, 129.4, 129.5, 130.3, 130.8, 130.9, 136.3, 173.0. HR-ESI-MS *m/z*: [M + H]$^+$ calcd. for 813.3248, found 813.3248. HPLC: t_R = 5.8 min over 15 min of eluent (90% CH_3OH and 10% H_2O), purity 96.7%.

S2.3. Fluorescence spectroscopy

Stock solution of **LKB**s (5 mM) was prepared in DMSO. Stock solutions of 0.5 mM of proteins were prepared in HCl-Tris buffer (0.05 M, pH 7.4). The fluorescence measurements were carried out with a path length of 5 mm and an excitation wavelength at 345 nm by scanning the spectra between 355 nm and 750 nm. The bandwidth for both excitation and emission spectra was 5 nm. Unless otherwise mentioned, all the spectra were recorded at 25 °C.

S2.4. Fluorescence imaging of cells

Cells were cultured in growth medium supplemented with 10% FBS. Cells (2.0 × 10^4/well) were seeded on a black 96-well microplate with optically clear bottom (Greiner bio-one, Germany) overnight. The cells were incubated with 20 μM of probe for 15 min. Then the cells on the microplate were rinsed in warm PBS and fixed by 4% paraformaldehyde for 20 min at room temperature. After three rinses in PBS the fluorescence was detected and photographed with an Operetta high content imaging system (Perkinelmer, US).

S2.5. Cell viability assay

Cells were cultured in DMEM supplemented with 10% FBS. Cells (5.0 × 10^3/well) were seeded on a 96-well plate (Corning, U.S.) overnight. After seeding, cells were maintained in growth medium treated at increasing concentrations (6.25 μM, 12.5 μM, 25 μM, 50 μM, 100 μM) of probes (dissolved in DMSO, final concentration) for 30 min. Then the medium was removed and replaced with fresh DMEM for 12 h at 37 °C. Then, 20 μL of MTS (Promega Corp) solution (2 mg mL^{-1}) was added to each well for 2 h at 37 °C. The absorbance was finally measured on a SpectraMax 340 microplate reader (Molecular Devices, USA) at 490 nm with a reference at 690 nm. The optical density of the result in MTS assay was directly proportional to the number of viable cells. Each experiment was done in triplicate.

S2.6. Confocal laser scanning microscopy

Hep-G2 cells were cultured in DMEM supplemented with 10% FBS. Cells (1.5 × 10^5/well) were seeded in a bottom-covered 24-well plate containing 0.2% gelatin (Corning, US) overnight. Early Endosomes-GFP BacMam 2.0, Cell Light® Early Lysosme-GFP, BacMam 2.0 (Life Technologies, US), GFP-rab11WT or GFP-rab7WT plasmid (Addgene) was transfected into Hep-G2 cells using Lipofectamine 2000 (Invitrogen, US) according to the manufacturer’s instructions. Forty-eight hours after transfection, the old medium was replaced with fresh medium containing 80 μM **LKB2** and incubated for another 2 h. Then the cells on the microplate were rinsed in warm PBS and fixed by 4% paraformaldehyde for 20 min at room temperature. After rinsing twice with PBS, syto63® red fluorescent nucleic acid stain (Invitrogen, US) (1 μM, 0.5 mL) was added to the cultures and incubated for 40 min at 37°C. After rinsing three times with warm PBS the fluorescence was detected and photographed with confocal laser scanning microscopy (Olympus, Japan).

S3. Additional references

1. Preparation of compound **1**: Jin, Z. *et al.* Clicking fluoroionophores onto mesoporous silicas: a universal strategy toward efficient fluorescent surface sensors for metal ions. *Anal. Chem.* **2010**, *82*, 6343–6346.
2. Preparation of compound **6**: Li, K.-B. *et al.* Hepatoma-selective imaging of heavy metal ions using a 'clicked' galactosylrhodamine probe. *Chem. Commun.* **2014**, *50*, 11735-11737.
3. Preparation of compounds **7** and **9**: He, X.-P. *et al.* Dynamic tracking of pathogenic receptor expression of live cells using pyrenyl glycoanthraquinone-decorated grapheme electrodes. *Chem. Sci.* **2015**, *6*, 1996-2001.
4. Preparation of compound **8**: Kong, N. *et al.* Carbohydrate conjugation through microwave- assisted functionalization of single-walled carbon nanotubes using perfluorophenyl azides. *Carboydr. Res.* **2015**, *405*, 33-38.

S4. Original spectral copies

^{1}H NMR of **2**:

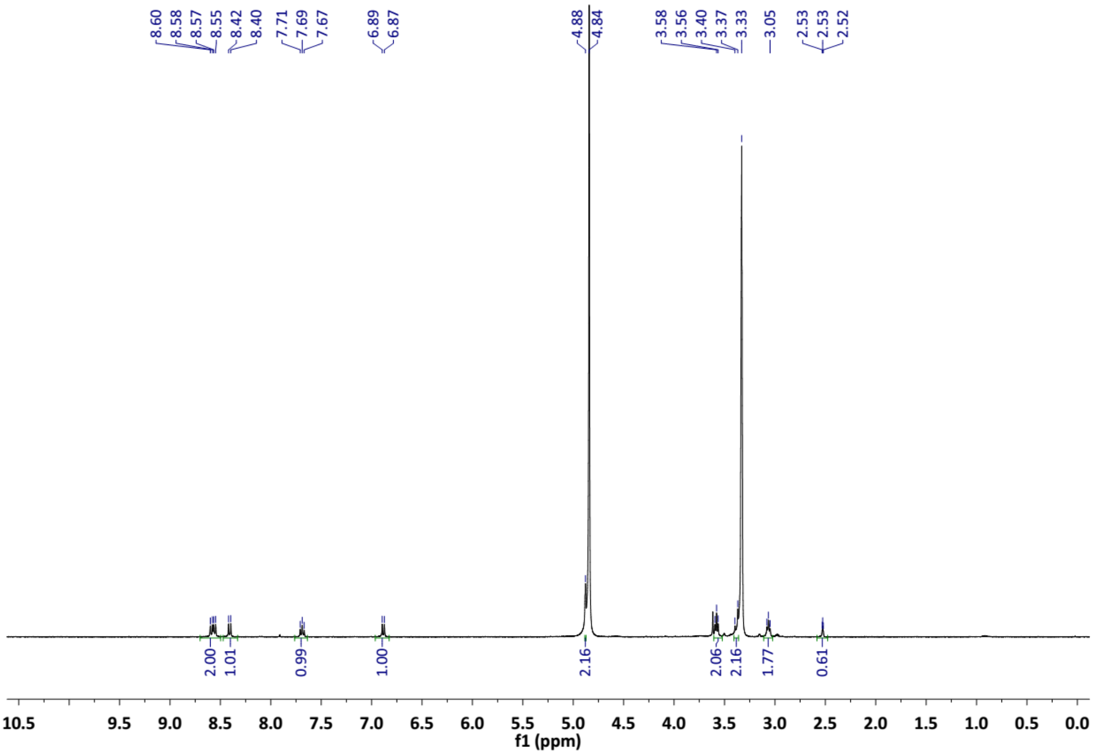

^{13}C NMR of **2**:

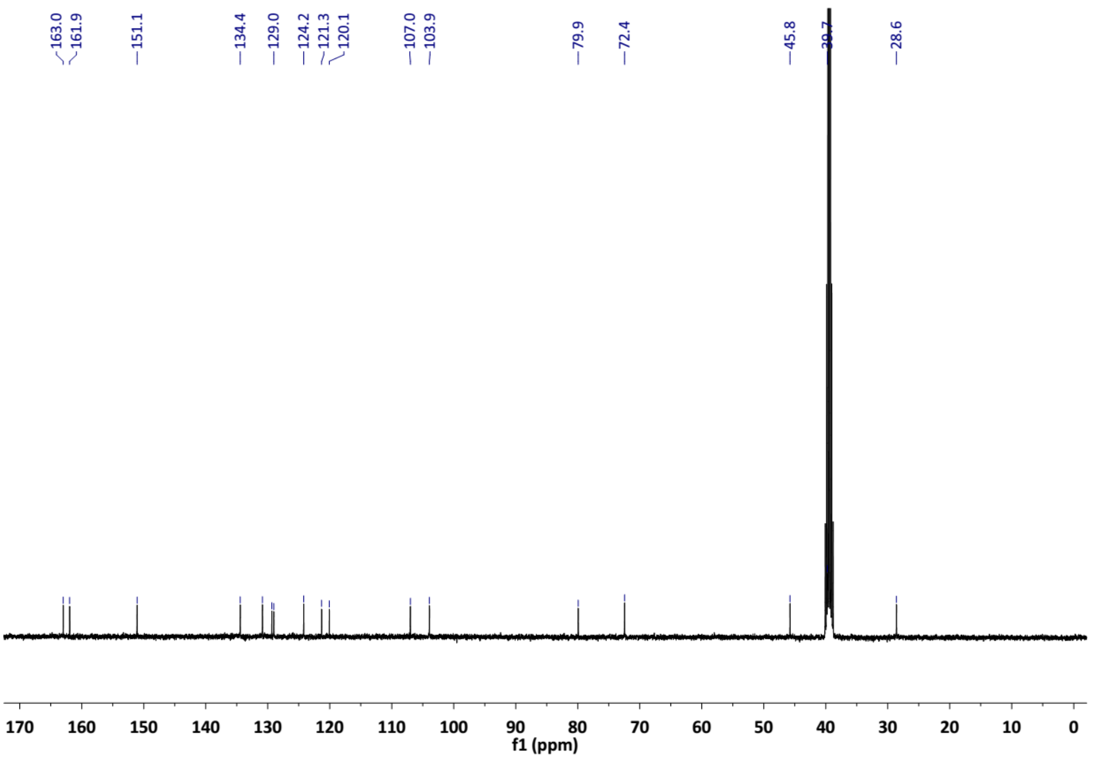

^{1}H NMR of **3**:

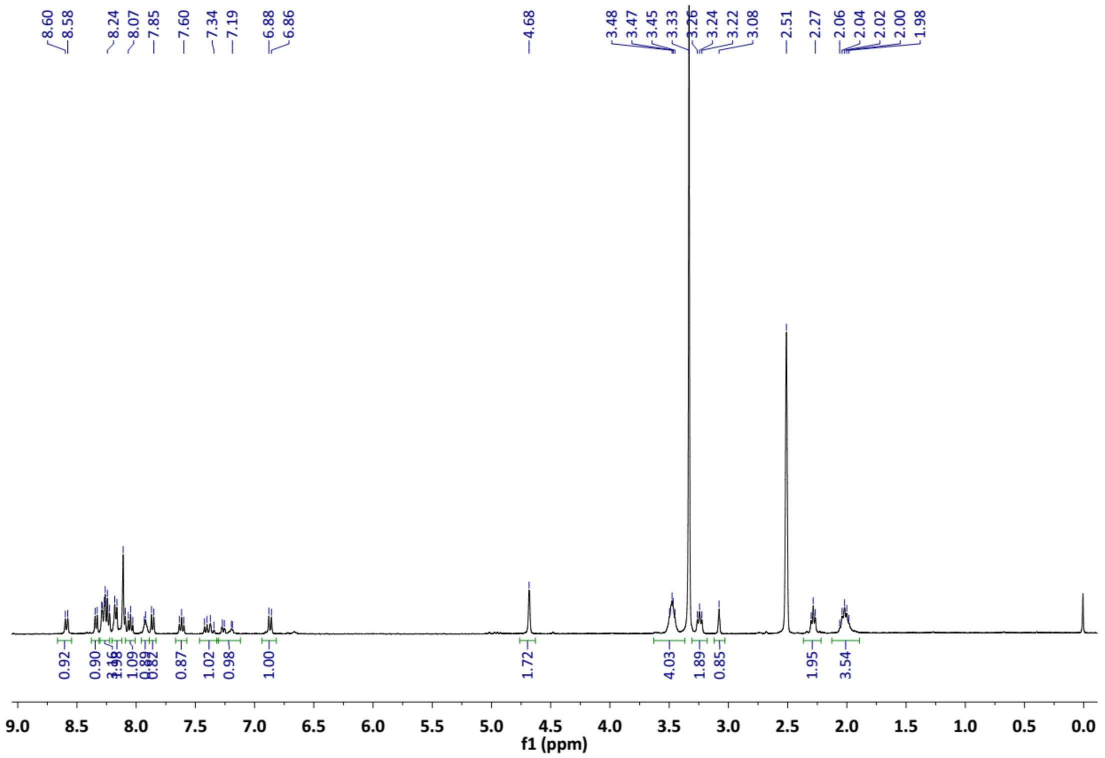

^{13}C NMR of **3**:

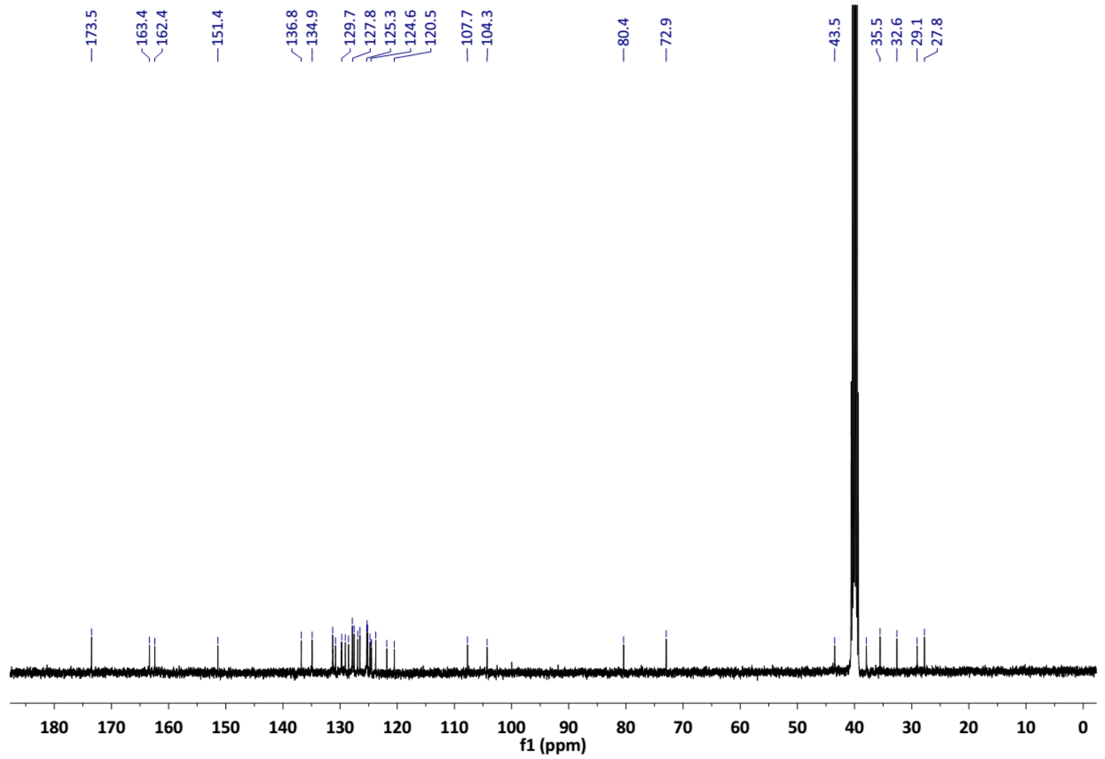

^{1}H NMR of **4**:

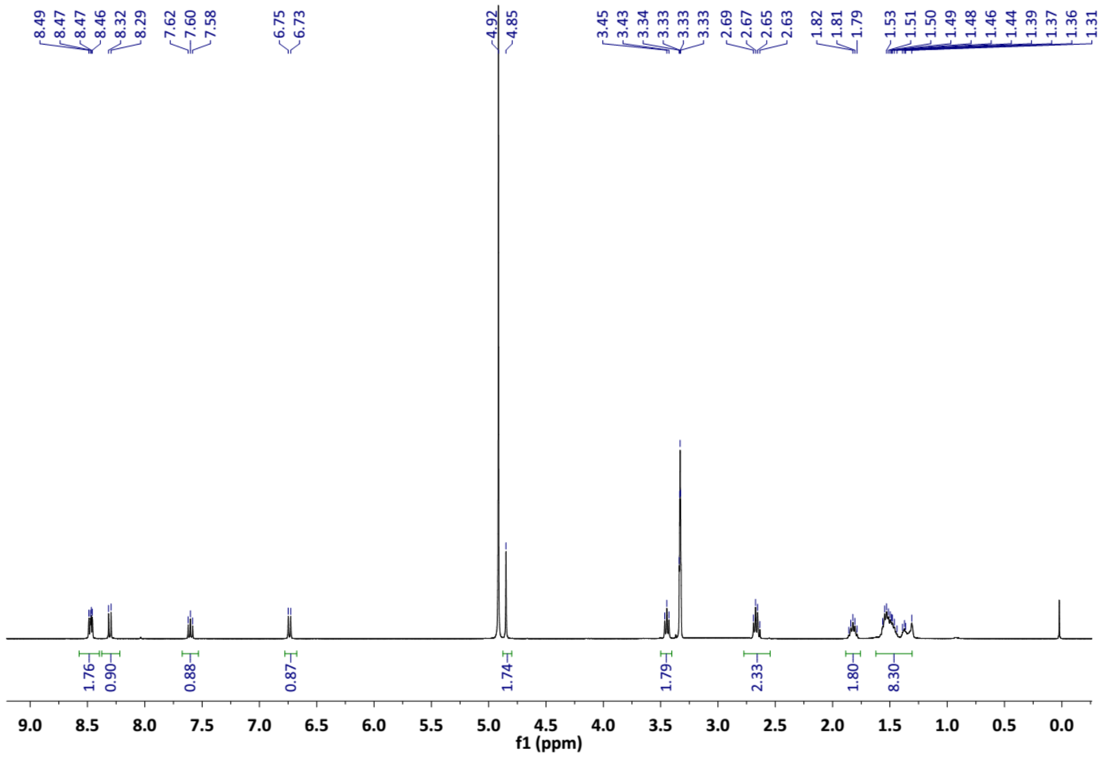

^{13}C NMR of **4**:

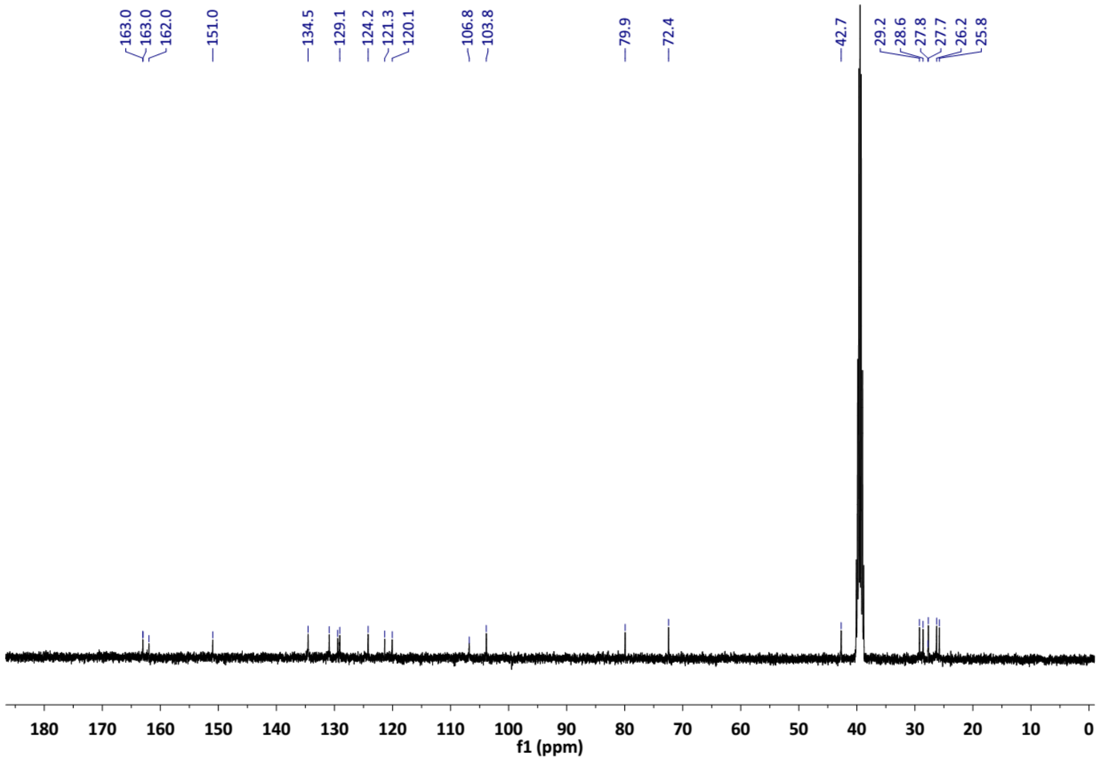

^{1}H NMR of **5**:

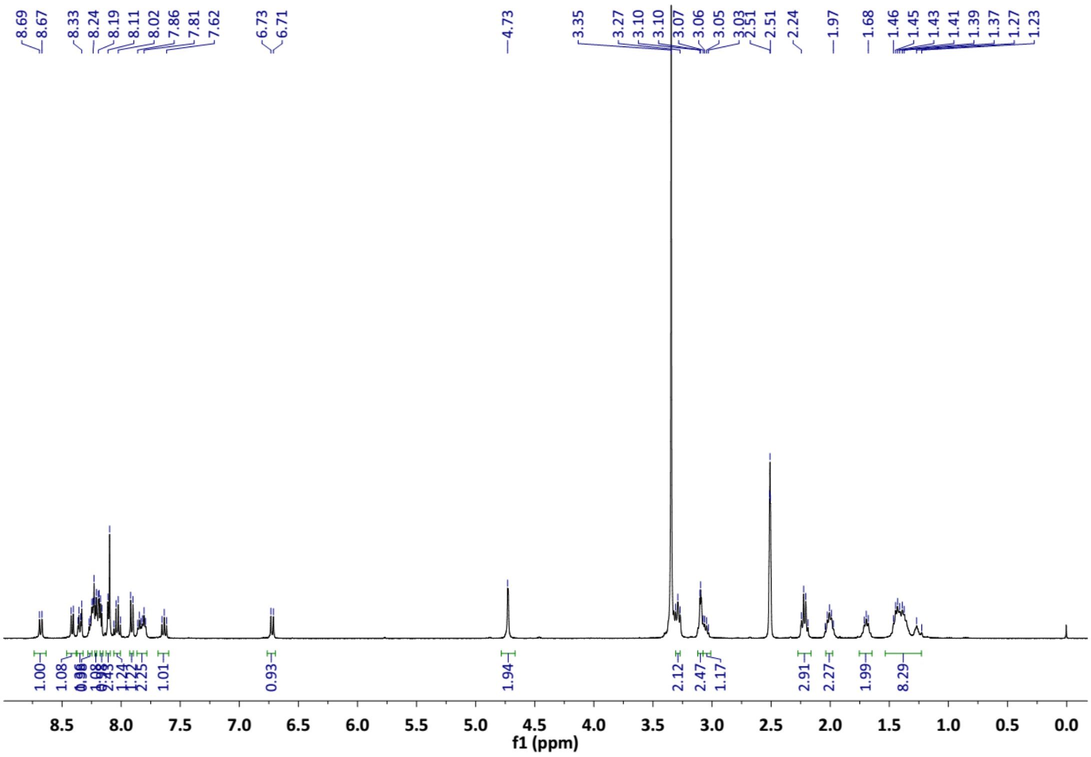

HR-ESI-MS of **5**:

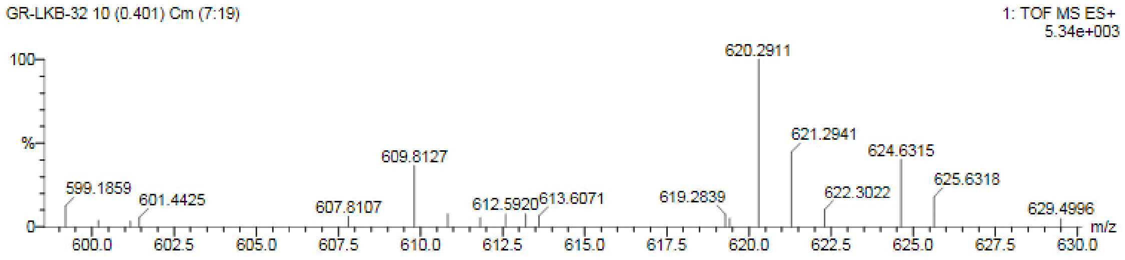

Minimum:				-1.5			
Maximum:		30.0	50.0	100.0			
Mass	Calc. Mass	mDa	PPM	DBE	i-FIT	i-FIT (Norm)	Formula
620.2911	620.2913	-0.2	-0.3	24.5	9.3	0.0	C41 H38 N3 O3

^{1}H NMR of **LKB1**:

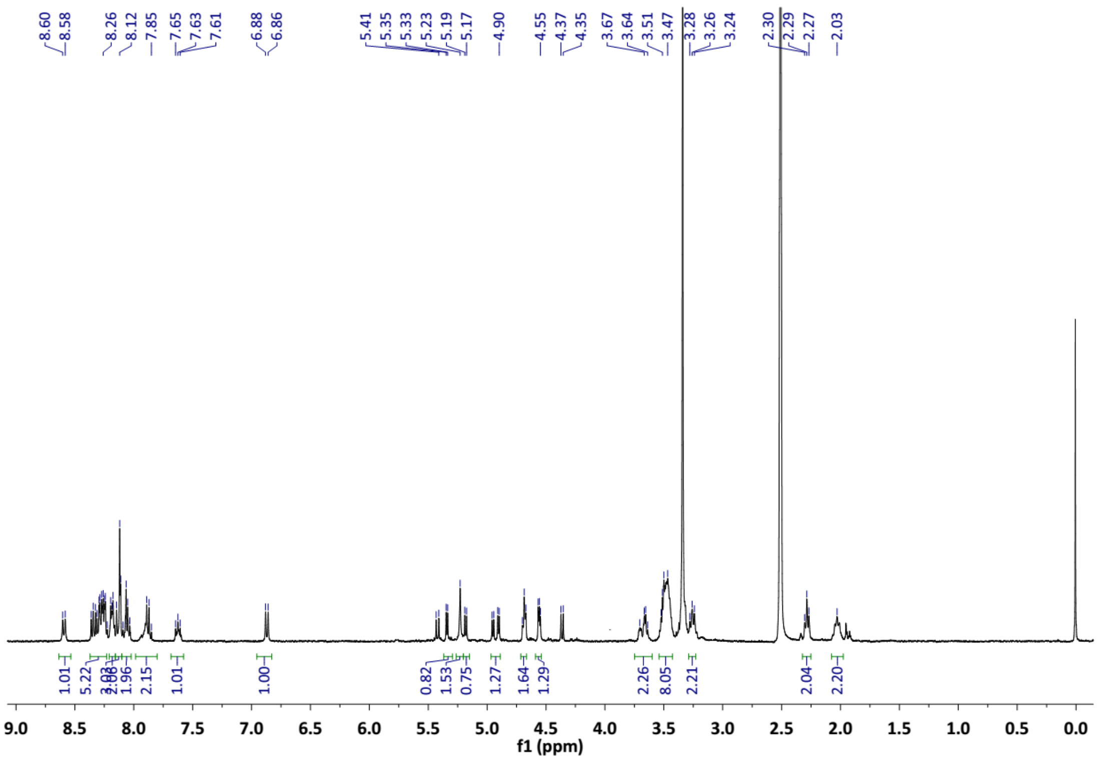

^{13}C NMR of **LKB1**:

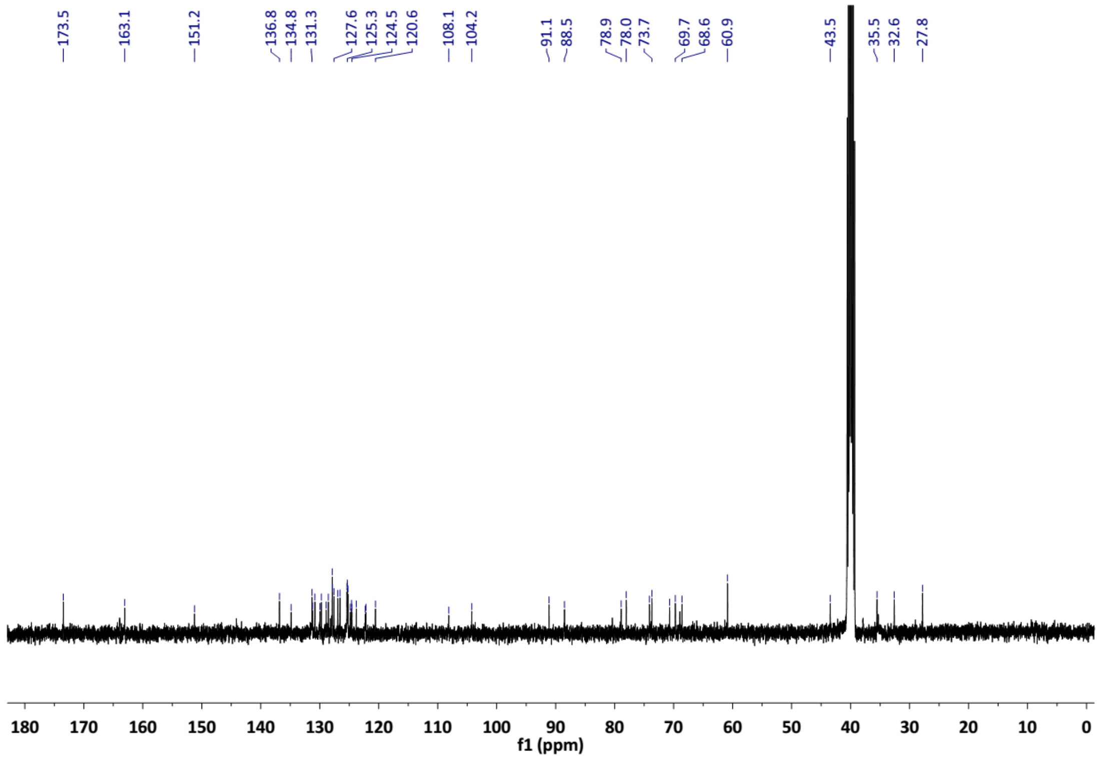

^{1}H NMR of **LKB2**:

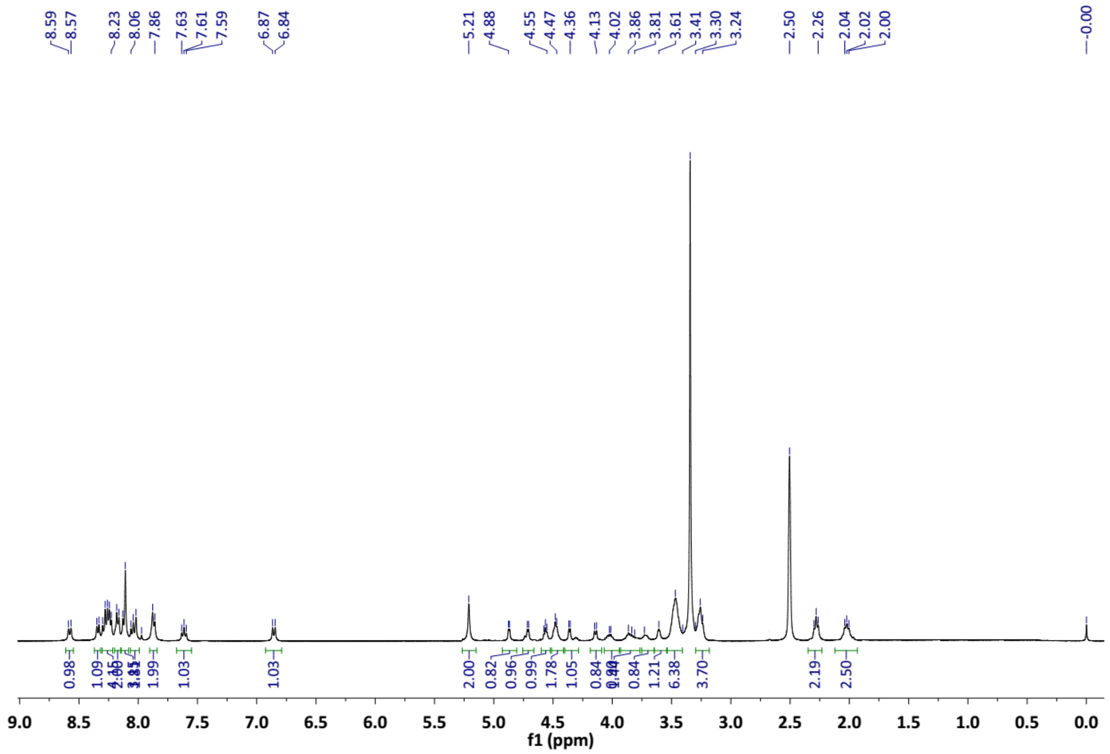

^{13}C NMR of **LKB2**:

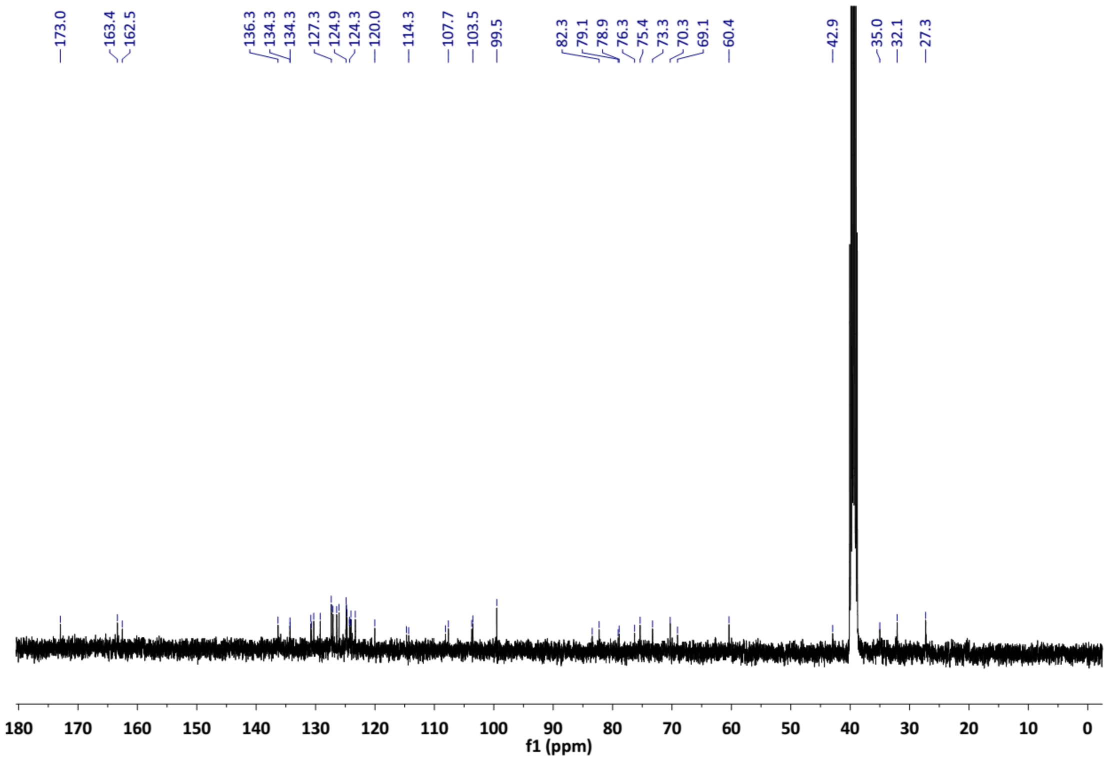

^{1}H NMR of **LKB3**:

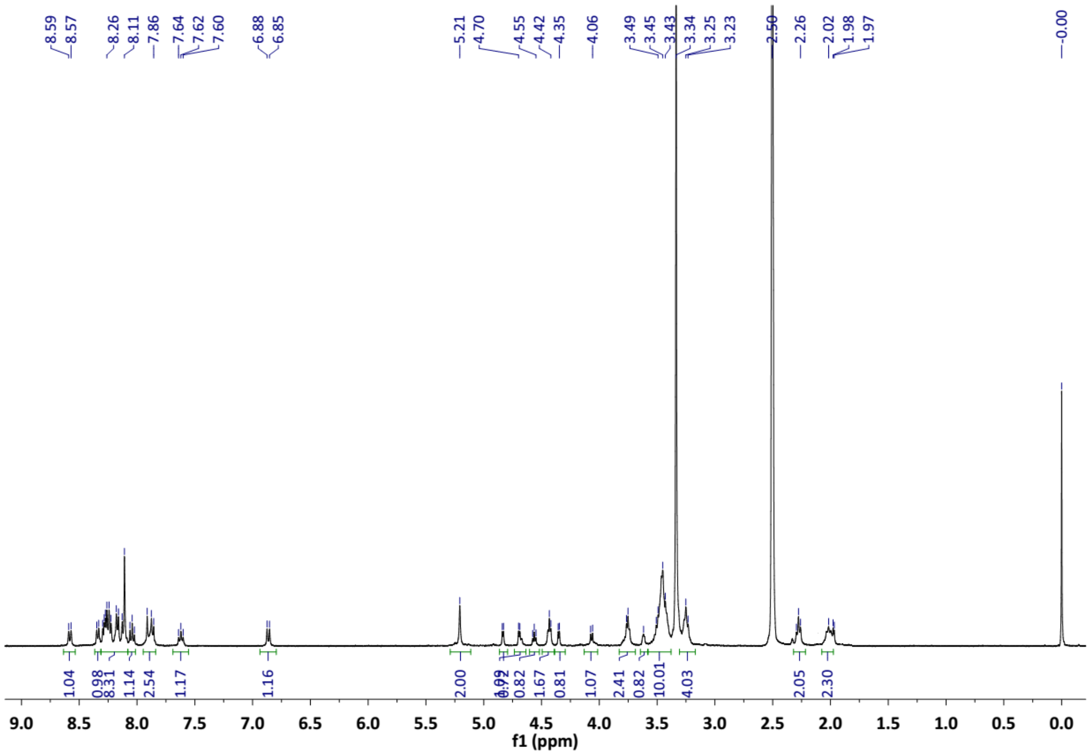

HR-ESI-MS of**LKB3**:

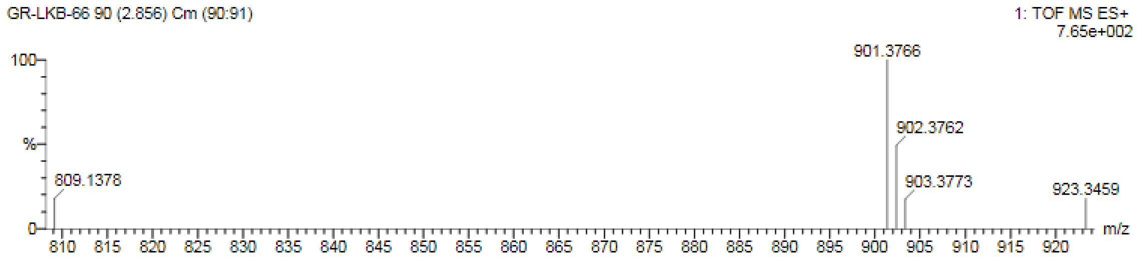

Minimum:				-1.5			
Maximum:		300.0	50.0	100.0			
Mass	Calc. Mass	mDa	PPM	DBE	i-FIT	i-FIT (Norm)	Formula
901.3766	901.3772	-0.6	-0.7	26.5	10.3	0.0	C49 H53 N6 O11

^{1}H NMR of **LKB4**:

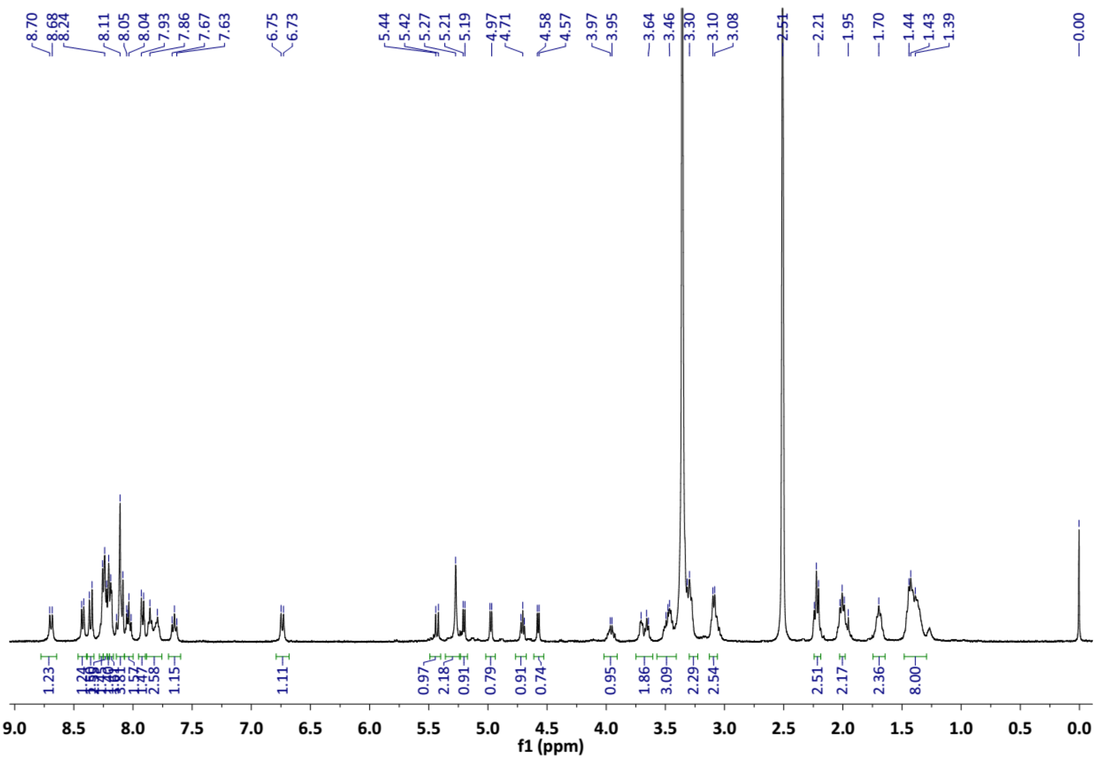

^{13}C NMR of **LKB4**:

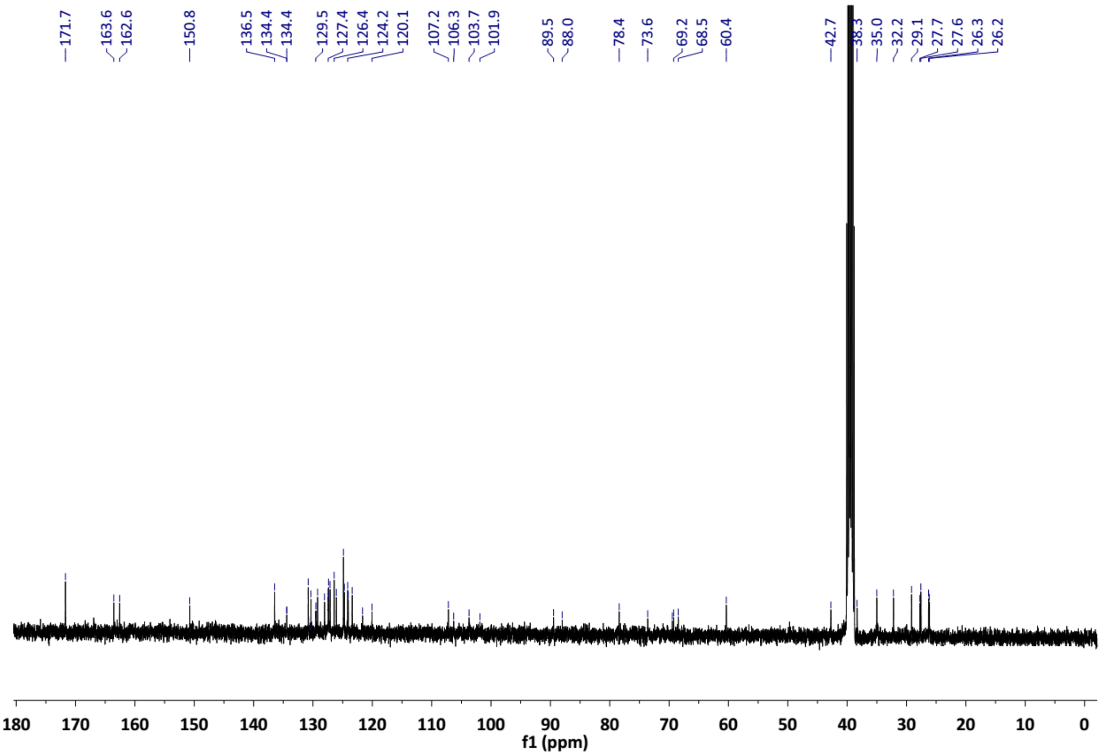

^{1}H NMR of **LKB5**:

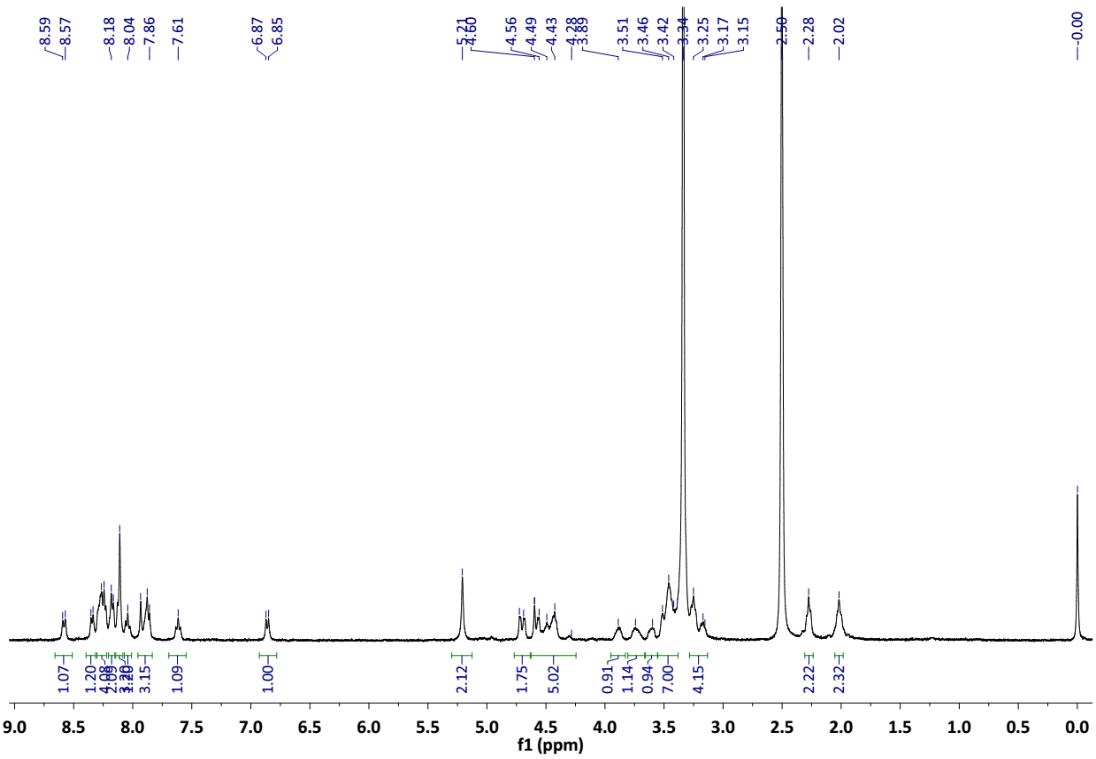

^{13}C NMR of **LKB5**:

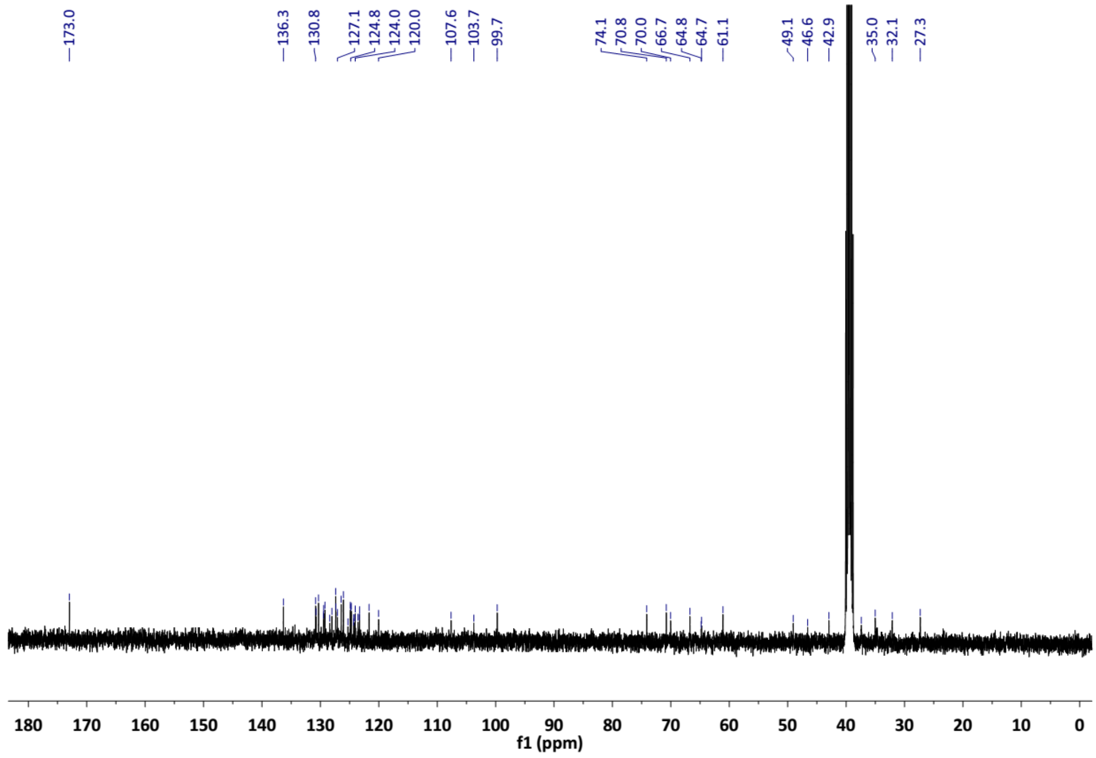

Supplement: Supplementary file 1 [file SC-007-C6SC02366E-s001.pdf]
